# A Mammary Organoid Model to Study Branching Morphogenesis

**DOI:** 10.3389/fphys.2022.826107

**Published:** 2022-03-16

**Authors:** Marika Caruso, Sjanie Huang, Larissa Mourao, Colinda L. G. J. Scheele

**Affiliations:** ^1^Laboratory for Intravital Imaging and Dynamics of Tumor Progression, VIB Center for Cancer Biology, KU Leuven, Leuven, Belgium; ^2^Department of Oncology, KU Leuven, Leuven, Belgium

**Keywords:** mammary gland, branching morphogenesis, 3D culture, mammary organoids, mammary gland development

## Abstract

Branching morphogenesis is the process that gives rise to branched structures in several organs, such as the lung, the kidney, and the mammary gland. Although morphologically well described, the exact mechanisms driving branch elongation and bifurcation are still poorly understood. Signaling cues from the stroma and extracellular matrix have an important role in driving branching morphogenesis. Organoid models derived from primary mammary epithelial cells have emerged as a powerful tool to gain insight into branching morphogenesis of the mammary gland. However, current available mammary organoid culture protocols result in morphologically simple structures which do not resemble the complex branched structure of the *in vivo* mammary gland. Supplementation of growth factors to mammary organoids cultured in basement membrane extract or collagen I were shown to induce bud formation and elongation but are not sufficient to drive true branching events. Here, we present an improved culture approach based on 3D primary mammary epithelial cell culture to develop branched organoids with a complex morphology. By alternating the addition of fibroblast growth factor 2 and epidermal growth factor to mammary organoids cultured in a basement membrane extract matrix enriched with collagen type I fibers, we obtain complex mammary organoid structures with primary, secondary, and tertiary branches over a period of 15–20 days. Mammary organoid structures grow >1 mm in size and show an elongated and branched shape which resembles *in vivo* mammary gland morphology. This novel branched mammary organoid model offers many possibilities to study the mechanisms of branching in the developing mammary gland.

## Introduction

The mammary gland is a highly dynamic organ with a branched morphology (Lu and Werb, 2008; Macias and Hinck, 2012). Throughout different developmental stages, the mammary gland undergoes drastic morphological transformations (Brisken, 2002; Oakes et al., 2008). One of these events occurs during puberty, during which a simple rudimentary tree develops into a complex branched system of ducts and lobules. This pubertal branching morphogenesis is orchestrated by rising levels of growth hormone and estrogen, which together induce a phase of extensive growth driven by specialized multilayered structures known as terminal end buds (TEBs; Sternlicht et al., 2006; Huebner et al., 2014). By regular bifurcation events and subsequent ductal elongation, TEBs give rise to the highly elaborate ductal system of the adult mammary gland filling the entire fat pad (Williams and Daniel, 1983; Hinck and Silberstein, 2005). Further remodeling occurs during multiple stages in the adult gland, such as estrous cycle-driven formation and regression of short tertiary branches or alveologenesis upon pregnancy, always accompanied by major morphological and functional changes of the mammary epithelium.

In the last two decades, research in the mammary field has mainly focused on dissecting the role of hormones, multiple growth factors, and stromal interactions during branching morphogenesis and adult remodeling. Although successful in deciphering multiple functional interactions, all these studies were restricted to characterization at one specific time point because of the limited availability of tools for optical monitoring of fast and dynamic events in the *in vivo* gland. Emerging *in vivo* techniques such as intravital imaging combined with advanced multiphoton microscopy have started to elucidate the dynamics and single cell contributions to these main remodeling events (Scheele et al., 2017; Dawson et al., 2021; Jacquemin et al., 2021; Messal et al., 2021). However, despite providing a valuable tool to follow the same mammary ducts over time, their application is still confined to a restricted time window, low-throughput, and limited by the need for technological expertise.

In parallel to emerging imaging technologies, efforts have been concentrated on *in vitro* organotypic systems of the mammary gland (Simian et al., 2001; Debnath et al., 2003; Ewald et al., 2008, 2012). Although multiple organoid models derived from different organs have achieved high levels of complexity and morphological reproducibility of *in vivo* structures, the complexity of murine mammary organoids is still limited (Clevers, 2016; Kim et al., 2021; Vazquez-Armendariz and Herold, 2021). Current mammary organoid culture protocols result in either bi-layered sphere-shaped organoids (referred to as cysts) or budding structures upon supplementation with different growth factors, such as FGF2, FGF7, FGF10, and Neuregulin1 (Fata et al., 2007; Nguyen-Ngoc et al., 2015; Jardé et al., 2016; Sumbal et al., 2020). Organoid culture methods using Matrigel with culture medium supplemented with FGF2 have provided valuable insights into the differentiation potential of mammary organoids (Jamieson et al., 2016). For instance, nanomolar concentrations of FGF2 are able to initiate budding in cystic organoids when cultured in Matrigel. These buds establish epithelial polarity and cell–cell interactions but are at the same time mostly devoid of myoepithelial cell coverage at the tips (Ewald et al., 2008). A mixture of Matrigel with collagen I was shown to increase bud elongation and improve coverage of the buds by myoepithelial cells, representing an important step toward a more physiological organoid model to study branching morphogenesis (Nguyen-Ngoc and Ewald, 2013). Although current mammary organoid culture methods recapitulate some key features of the mammary gland, including a bi-layered morphology, the “branched structures” are still far from reproducing the level of elongation and branching complexity that is observed in the *in vivo* network of interconnected primary, secondary (and tertiary) ductal structures.

Here, we report a novel mammary organoid culture protocol that results in complex branched structures *in vitro*. By combining mixed BME:collagen I (7:3 ratio) gels (referred to as 7B3C) with a timed addition of FGF2 and EGF, mammary spheres undergo multiple branching and elongation events resulting in high-level branched 3D mammary organoids. We demonstrate that the alternated addition of FGF2 and EGF results in mammary organoids with a superior morphology and complexity compared to the standard culture conditions with FGF2 or EGF addition alone. Primary mammary gland organoids cultured in 7B3C gels in combination with FGF2/EGF alternation treatment do not only result in a high organoid complexity with branches up to the fourth level but also exhibit large-scale elongation with branch lengths >0.5 mm. Altogether, our branching protocol can fill the gap in available *ex vivo* models and provides novel insights in branching morphogenesis.

## Materials and Methods

Building upon previously published guides reporting the mammary organoid assay (Ewald et al., 2008; Nguyen-Ngoc and Ewald, 2013; Nguyen-Ngoc et al., 2015), the procedures below describe an optimized method to obtain mammary epithelial organoids with a branched morphology which closely resemble the *in vivo* structure of the mammary gland. In addition, we provide a guide for multiphoton organoid imaging to follow organoid branching dynamics over multiple weeks. Finally, we include a description of several parameters to analyze organoid branching complexity.

### Isolation of Primary Mammary Epithelial Organoids

**Figure 1:**
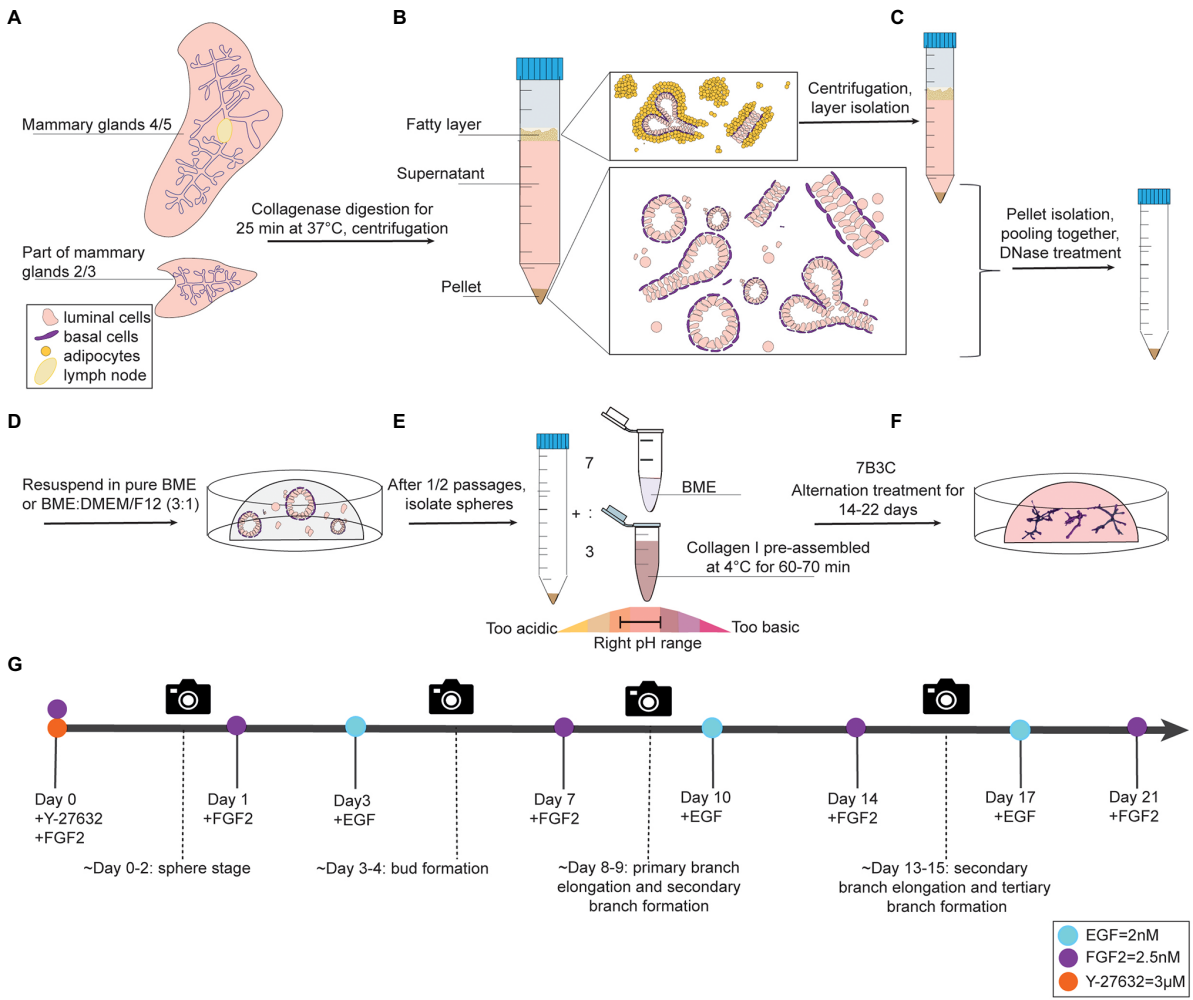
Organoid culture protocol and timeline. **(A-C)** Dissection, digestion, and centrifugation steps to isolate mammary epithelial fragments for organoid culture. **(D)** Organoid expansion step in BME to generate mammary spheres. **(E)** Collagen pre-assembly, pH adjusting step, and generation of BME:collagen I mixture (7:3) to plate mammary spheroids in 7B3C. **(F)** Alternation treatment with FGF2 and EGF to generate branched mammary organoids. **(G)** Timeline of mammary gland harvesting procedure and processing steps to obtain branched organoids in 7B3C gels using alternating treatment conditions.

1. Anesthetize mice using 3–5% isoflurane and euthanize by cervical dislocation. Next, make a midline incision and separate the skin from the peritoneum. Excise lymph nodes from both fourth glands and collect thoracic and inguinal mammary glands (2/3 and 4/5), as previously described (Nguyen-Ngoc et al., 2015). To reduce muscle contamination in the preparation, avoid harvesting of the portion of mammary gland 2/3 close to the chest wall muscles (Figure 1A, Supplementary Figure S1A).
2. Mince the pooled mammary glands with a scalpel to approximately 1 mm^3^ pieces in a sterile hood, and digest in *collagenase solution* for 30 min at 37°C while shaking at 180 rpm using an orbital shaker.
3. Centrifuge the tube for 10 min at 1,500 rpm to obtain a pellet containing mammary epithelial cells and fragments. The supernatant is composed of a lower aqueous layer and a floating “fatty layer” on the top. The “fatty layer” on top contains not only of fat tissue but also epithelial fragments trapped within the adipocytes (Figure 1B).
  3.1 Additional step: Isolate the fatty layer, digest it in 10 ml of *collagenase solution* for 30–40 min (180 rpm, 37°C) and centrifuge to derive a second pellet. This step allows considerably higher yield of organoid/epithelial pieces.
    - Critical step: the fatty layer will stick to plastics or metals; for its isolation, it is advised to use 2.5% BSA-coated 1 ml pipette tips with a cut end. Aspirate not only the fatty layer, but also a part of the underlying aqueous layer (facilitating further fat aspiration).
4. Pool the pellets obtained from the two digestion steps and treat with 2 U/ml of DNase I in 4 ml of DMEM/F12 by gently shaking the tube by hand for 5 min (Figure 1C). Add 6 ml of DMEM/F12, mix the tube thoroughly and centrifuge at 1,500 rpm for 10 min.
  4.1 Additional step: if the pellet still shows consistent contamination by erythrocytes after centrifugation, remove the supernatant and treat the pellet with 1 ml of Red Blood Cell Lysing Buffer for 1 min, resuspend in 9 ml of DMEM/F12, and centrifuge again at 1,500 rpm for 10 min.
    - Note: from this step onward, whenever in contact with mammary epithelial cells, 2.5% BSA pre-coated tips are highly recommended to prevent cells from sticking to the plastic resulting in loss of material (Nguyen-Ngoc et al., 2015).
5. Inspect the pellet (10–20 μl) under the microscope to define its digestion level. If the pellet is mainly composed of small fragments/single cells and a limited number of epithelial pieces, proceed directly with plating. Instead, if the pellet is mainly composed of larger intact epithelial pieces, it is still under-digested (Supplementary Figure S1A,B). In this case, proceed with an additional step of enzymatic digestion that will increase the yield of small fragments/single cells from the remaining undigested epithelial pieces (Supplementary Figure S1C).
  5.1 For enzymatic digestion, remove the supernatant and add 1 ml of phenol red-free TrypleE^™^ Express to the pellet. Incubate for 7–12 min at 37°C. The incubation time with TrypleE^™^ can be adjusted depending on the level of under-digestion after *collagenase* treatment. However, this step has to be carefully monitored to avoid pellet over-digestion (Supplementary Figure S1D). Next, add 2 ml of FBS to stop the reaction, mix gently and centrifuge at 1,500 rpm for 10 min.
  5.2 Inspect the pellet again under the microscope. If the level of digestion is acceptable but some large epithelial pieces are still present, it is recommended to proceed with mechanical dissociation (Supplementary Figure S1E). To this end, add 200 μl of 2.5% BSA to the isolated pellet and pipet up and down for 50–60 times with a P200 pipette. A combination of enzymatic and subsequent mechanical dissociation is often needed to fully disrupt tissue pieces into small fragments.
  5.3 Additional step: to increase organoid yield, isolate the big epithelial fragments in a separate falcon tube using a pipette with a 2.5% BSA-coated tip (fragments will stick easily to the plastic of the tip) and enzymatically disrupt them further.
6. Add DMEM/F12 to the tube and centrifuge at 1,600–1,700 rpm for 10 min. Differential centrifugation steps as previously described (Nguyen-Ngoc et al., 2015) are omitted here to prevent organoid loss.

### Organoid Plating in BME

1. Remove the supernatant and resuspend the organoid pellet in BME, either in pure BME or in a BME:DMEM/F12 mix with a 3:1 ratio.
2. Plate the BME:mammary epithelial cell suspension in droplets of 30 μl in a 37°C pre-warmed 24-well plate (Figure 1D, Supplementary Figure S1C). Critical step: perform the first plating in no more than 5–6 BME droplets per animal, especially in presence of many single cells in the pellet. Seeding the isolated mammary epithelial cells too sparsely will drastically reduce the final organoid yield.
3. Swiftly flip the plate in a circular motion and incubate upside-down for 40 min at 37°C and 5% CO_2_ to allow BME polymerization. Plate and flip as fast as possible in order to avoid epithelial cell seeding on the bottom of the droplet resulting in 2D growth.
4. Gently add 650 μl of pre-warmed Y-BOM to the wells when polymerization time has elapsed.
5. Change to BOM without Y-27632 after 24–48 h and refresh medium every 3 or 4 days.

### Organoid Passaging

1. To passage organoids, add 1 ml of 1X ice-cold PBS and scrape the BME droplets with a P1000 tip to fasten matrix depolymerization.
2. Centrifuge at 1,500 rpm for 10 min and remove the supernatant without disturbing the cloudy layer of BME.
3. Incubate the pellet and cloudy layer for 30–40 min in ice to reduce BME viscosity and release organoids trapped in the matrix, thereby preventing organoid loss at each passage. This step can be skipped when performing an additional enzymatic dissociation step, as the cocktail of enzymes will degrade most of the remaining BME.
  3.1 If numerous epithelial pieces or big-size organoids are still present, perform an additional step of enzymatic dissociation and/or mechanical dissociation at this point. For enzymatic digestion, remove the supernatant and add 1 ml of phenol red-free TrypleE^™^ Express to the pellet. Incubate for 5–10 min at 37°C. Next, add 2 ml of FBS to stop the reaction, mix and centrifuge at 1,500 rpm for 10 min. For mechanical dissociation, add 200 μl of 2.5% BSA to the pellet and pipet up and down for 50–60 times with a P200 pipette.
4. Centrifuge at 1,500 rpm for 10 min, remove the supernatant and resuspend the pellet in BME. It is advised to use a split ratio of 1:2 (Supplementary Figure S1F).
5. Continue with plating as described in section “Organoid Plating in BME”.

### Preparation of Pre-assembled Collagen I

Collagen I is commercialized as an acid-solubilized solution purified from rat tails. The solution needs to be neutralized to allow gelation. For collagen I preparation and assembly to derive BME:collagen I mixed gels, the main steps described by Nguyen-Ngoc et al. (2015) were followed with some adaptations to allow for more flexibility in terms of pH range adjustments and a reduced time to reach neutrality, thereby improving the quality of collagen assembly. During the neutralization step, when the pH changes from acidic ➔ neutral ➔ basic, the color of the solution changes from light green/yellow ➔ light pink/salmon ➔ dark pink, respectively (Figure 1E).

- Note: all the following steps need to be performed on ice, including mixing after each addition.

1. To prepare 1 ml of neutralized collagen I, first combine 864 μl [stock (C) = 3.81 mg/ml, final (C) = 3.29 mg/ml] of collagen I (acidic) with 5 μl of 0.5 N NaOH (basic) and mix well. At this point, the solution will still be transparent and has a basic pH (because of the relative amount of acid–base solutions in the mixture). DMEM addition in following steps allows to precisely adjust the pH to neutrality.
2. Add 80 μl of 10X low glucose DMEM (acidic, the pH is lowered by the addition of DMEM) and mix thoroughly. The solution will turn pink (Figure 1E).
3. Add 10 μl of 10X low glucose DMEM and mix. The pH is lowered by DMEM and the solution will turn into a lighter pink at each DMEM addition (Figure 1E, light pink corresponds to a pH ~ 8).
4. Use 10 μl of 10X low glucose DMEM to adjust the pH to neutrality by adding 4–5 μl per time.
  - Critical step: mix thoroughly after each addition until the color remains stable in all parts of the solution since even few microliters of “excessive” DMEM can turn the slightly basic solution directly into acidic, when the pH approaches the neutral range. The desired color is light salmon which correspond to a pH of 7.1–7.5 (Figure 1E).
5. When the solution turns light pink, add 2–5 μl of 10X low glucose DMEM to turn it into a very light pink-salmon color. This step ensures that the final pH is not slightly basic (Figure 1E). The pH can be measured using pH strips.
6. If the solution remains dark pink after addition of 100 μl of 10X low glucose DMEM, continue adding 10X low glucose DMEM in small amounts (Figure 1E). Instead, if the collagen I solution turns light yellow (slightly acidic) the addition of 0.5 μl of NaOH (or dipping the tip in NaOH solution and then in the collagen I solution) allows to adjust the pH to neutral.
  - Note: Prior to the collagen I pre-assembly step, coat all culture wells with a thin layer of neutralized collagen to facilitate the attachment of the BME:collagen I gel to the bottom of the well and incubate the coated plate at 37°C until subsequent plating steps (Nguyen-Ngoc et al., 2015).
7. Incubate the neutralized collagen I solution at 4°C for pre-assembly. Best organoid structures in our protocol were achieved with a pre-assembly time of 65 min. After this time, the collagen I solution will turn cloudy and fibrous, and it is referred to as pre-assembled collagen I.
  - *PAUSE POINT* (60–70 min): during this time, harvest the previously seeded organoids from the BME drops in order to have a ready-to-plate organoid pellet when the collagen assembly time has elapsed.
  - Note: The properties of collagen I gels vary depending on multiple factors during preparation, including temperature, pH, and collagen concentration. For this reason, it is important that the tube containing the neutralized collagen I solution is well inserted in ice for the entire time of pre-assembly to keep the temperature constant.

### Mammary Gland Organoid Culture in 7B3C Gels Under FGF2/EGF Alternation Treatment

Note: All these steps have to be performed keeping BME, collagen I, and the mixed matrix on ice.

1. Prepare a 7B3C gel by mixing 7 parts of BME and 3 parts of pre-assembled collagen I (Figure 1E).
2. Gently pipet up and down 40–50 times with a P200 pipette to obtain a homogenous BME:collagen I solution.
3. Add the BME:collagen I mix to the organoid pellet and mix well.
  - Crucial step: remove the supernatant (DMEM/F12) above the organoid pellet completely before adding the BME:collagen I mixture to prevent dilution of the assembled matrix with leftover medium leading to a change in matrix stiffness.
  - General note: Pipette up and down to well resuspend the organoids within the BME:collagen I mixture prior to plating. These steps are fundamental to have a homogenous solution and, as a consequence, isotropic gels for branching.
4. Plate the mixed matrix/organoid suspension in droplets of 30 μl on the collagen I pre-coated well bottoms. Use glass bottom plates for multiple time point imaging on a confocal microscope and/or subsequent immunofluorescent staining.
5. Gently add 650 μl of pre-warmed *Basic Organoid Medium* supplemented with 3 μM Y-27632 and 2.5 nM FGF2 (YF-BOM; Figures 1E,F).
6. The next day, replace the YF-BOM medium with F-BOM (BOM supplemented with 2.5 nM of FGF2).
7. Replace the F-BOM medium at day 3 after plating with E-BOM. Keep on repeating this medium exchange with F-BOM and E-BOM every fourth and third day, respectively (Figure 1G). On day 3/4 after plating, the first bud structures will appear, and 8/9 days after plating the first primary and secondary branches will form. The experiment can be continued until the right organoid complexity has been reached or until the 7B3C gel starts to disintegrate.

### Tracking Individual Organoids for Multiple Timepoint Imaging

**Figure 2:**
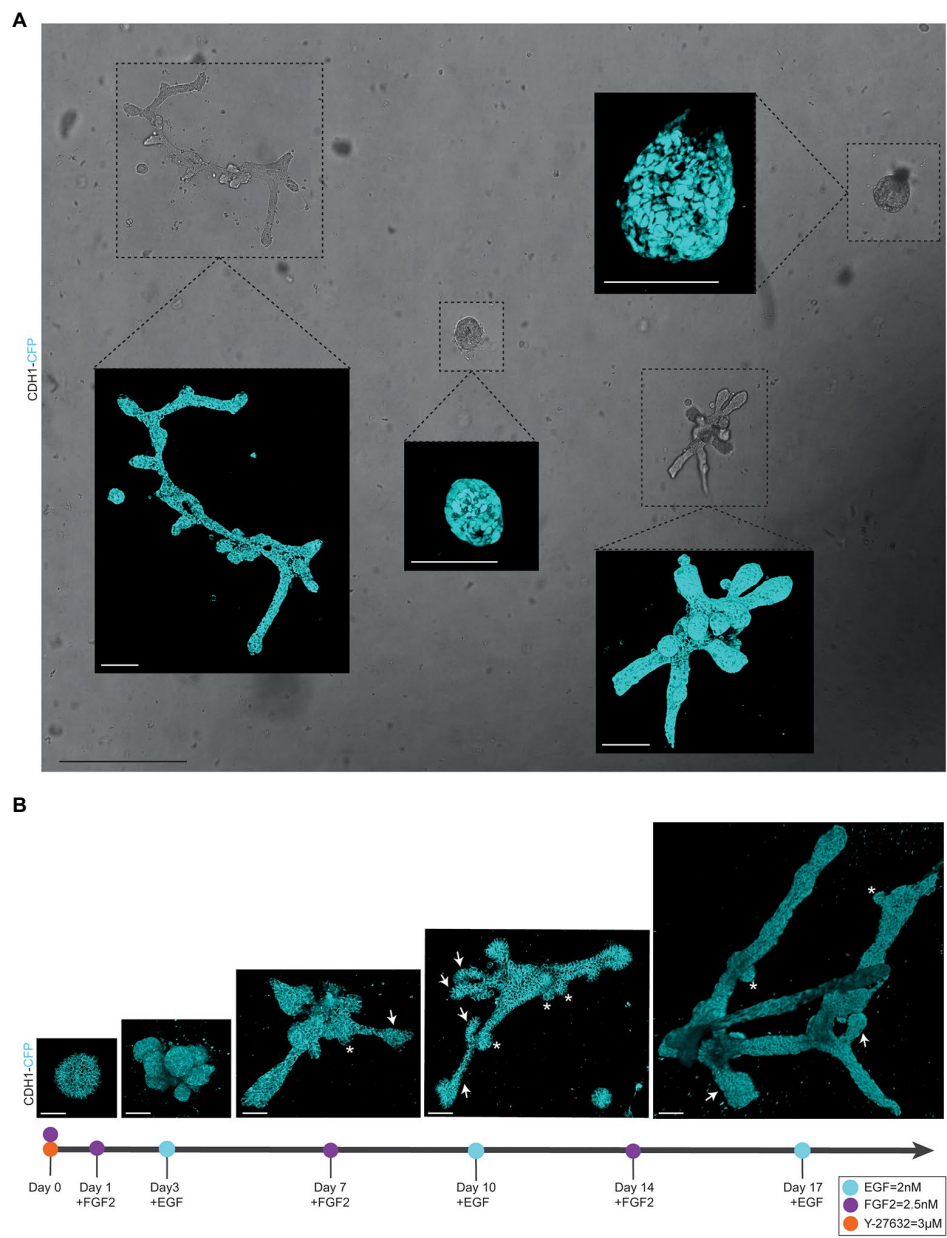
Alternating culture conditions in 7B3C gels result in branched mammary organoids. **(A)** Overview scan of mammary spheres and branched organoids used to retrace the same organoids over time based on their location within the culture well (brightfield image). Insets depict confocal images of *Cdh1-CFP* signal (cyan) of the individual organoids (3D rendering). Scale bars represent 500 μm for the brightfield image and 100 μm for the confocal images. **(B)** Representative 3D multiphoton images of *Cdh1-CFP* (cyan) organoid structure over time during the main morphogenetic changes occurring in 7B3C under alternation treatment. Budding structures (<70 μm) are indicated with white asterisks; branches (>70 μm) are indicated with white arrows. Scale bars represent 50 μm.

To follow branching morphogenesis during FGF2/EGF alternation treatment, the mammary organoids can be imaged using confocal imaging. Typically, when following the suggested organoid branching timeline (Figure 1G), the organoids should be imaged every 3/4 days to enable detailed analysis of morphological changes and branching dynamics. To follow the same organoids over time, it is crucial to obtain an overview of each well and identify the same organoids based on structure and location within the well (Figure 2A). To this end, we made use of the spiral function in the LasX Navigator software (Leica Microsystems). A central Z-position was selected so that most organoids were visible. If needed, when a single Z-layer does not capture all organoids, overview scans set at different Z-positions can be taken. Next, organoids of interest were selected to generate detailed Z-stacks. E-cadherin CFP was excited by 2-photon imaging using an Insight X3 laser (680–1,300 nm wavelength range, Spectra-Physics) set at 860 nm and detected in between 455 and 490 nm. Organoids were imaged at different time points of alternation treatment using a Z-step size ranging from 0.5 to 3.0 μm (Figures 2A,B). All images were captured in 12 bit, 600 Hz, 1,024 × 1,024 pixels and acquired with a 25X water objective.

### Organoid Immunofluorescent Staining

To perform immunofluorescent staining in 3D cultures:

1. Remove organoid medium from the wells. All the following steps are performed by adding solutions directly on top of the 7B3C dome in the wells.
  - Note: gently remove and add solutions from the wells with a P200 pipette to prevent droplet detachment.
2. Fix samples with 4% PFA for 10–15 min at room temperature on an orbital shaker at 25 rpm.
3. Remove PFA and wash 3× 10 min with 1X PBS. If not used immediately, seal the plate with parafilm and store in the fridge at 4°C up to 1 month.
4. For permeabilization and blocking, incubate with permeabilization buffer (5% normal goat serum and 0.5% Triton X-100 in 1X PBS) for 2–4 h at room temperature, or overnight at 4°C (on an orbital shaker at 25 rpm).
5. Remove the blocking solution, add the primary antibodies in blocking buffer (5% normal goat serum in 1X PBS) at the desired dilution and incubate overnight at 4°C.
6. Remove the primary antibody solution and wash 3 × 15 min with 1X PBS (on an orbital shaker at 25 rpm).
7. Add secondary antibodies at the desired dilution and incubate for >5 h at room temperature covered in aluminum foil (on an orbital shaker at 25 rpm).
  - Optional: for nuclear staining, add DAPI solution (1:500) to the wells and incubate for 2 h at room temperature.
8. Remove secondary antibody (and DAPI) solution and wash 3 x 15 min with 1X PBS.
9. Mount samples with Vectashield antifade mounting medium by adding ~3 droplets per well to cover the droplet.
10. Store samples at 4°C protected from light until imaging.

### Imaging of Stained Organoids

Stained organoid and whole-mount samples were imaged using an inverted confocal microscope (SP8 Leica Microsystems). In stained whole-mount organoids, endogenous Ecad-CFP was excited by 2-photon imaging using an Insight X3 laser set at 860 nm and detected in between 455 and 490 nm. SMA (Alexa647 secondary antibody) was excited at 635 nm and detected between 660 and 700 nm. Whole-mount mammary gland preparation was performed as previously described (Scheele et al., 2017). Mammary glands were stained for E-cadherin and SMA, and imaged as follows: E-cadherin (Alexa-488 secondary antibody) was excited at 488 nm and SMA (Alexa-647 secondary antibody) at 635 nm, and detected between 410 and 450 nm and 660 and 700 nm, respectively.

### Analysis of Branching Parameters

**Figure 3:**
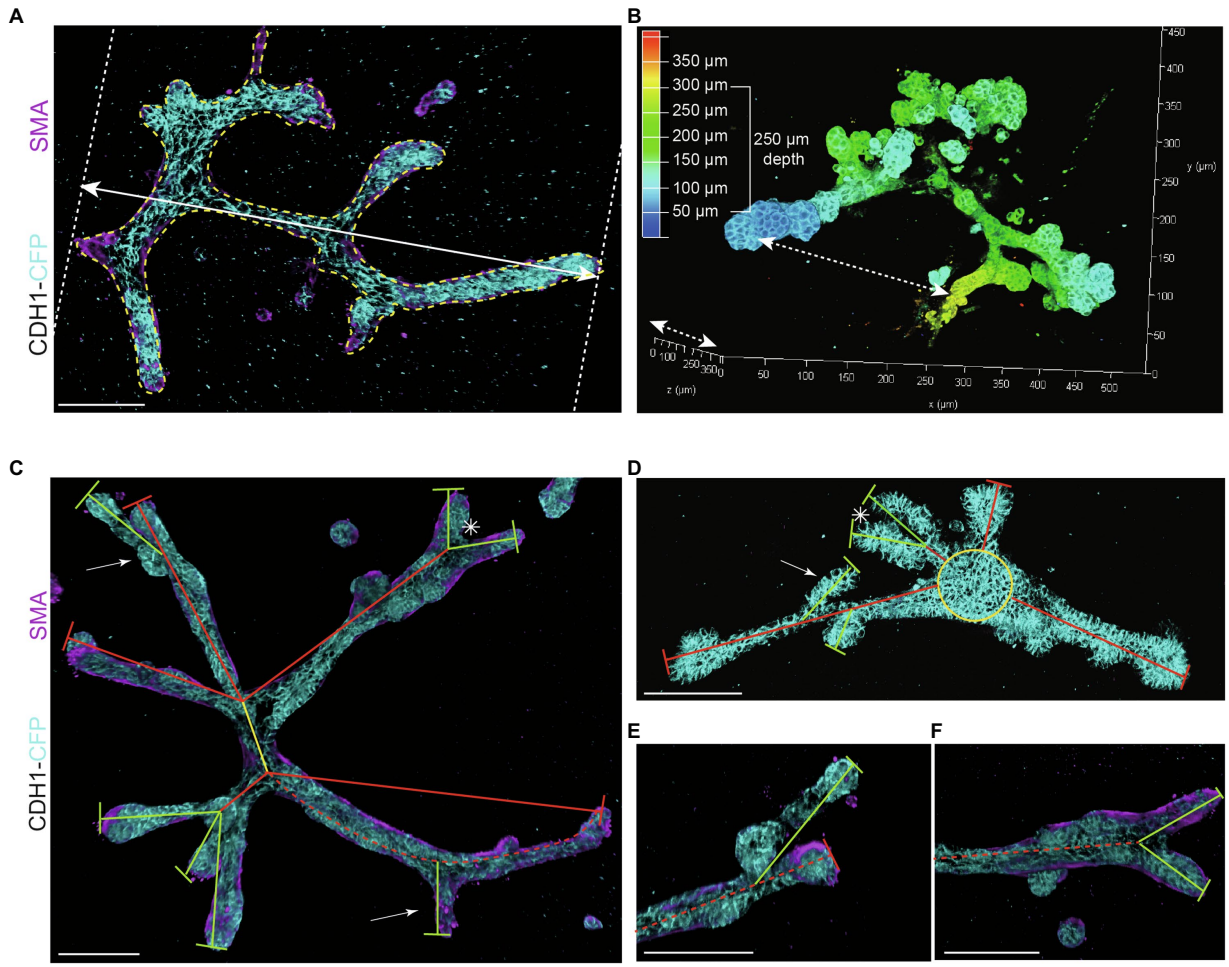
Measurements to describe branched organoid morphology. **(A)** To determine organoid area, the borders of branched organoids were marked (yellow dotted line) using maximum intensity projections and the size of the yellow marked area was determined. Organoid length was measured in the direction of branch elongation (white arrow). **(B)** Organoid depth was measured using the depth coding function in LasX 3D visualization software (Leica Microsystems) or derived from the color scheme of the depth scale bar. **(C)** Representative 3D rendering of a branched organoid lacking a central core after elongation and branching. The primary branch was defined as the center of the organoid (yellow line), connected to secondary branches (red lines) and tertiary branches (green lines). Bifurcation point is indicated with asterisk; side branches/buds are indicated with white arrows. Branch length was calculated as the distance between two branch points or the distance between branch tip and previous branch point. **(D)** Representative 3D rendering of an organoid partially retaining a central core (yellow) after elongation. Primary branches are indicated with red lines, and secondary branches are indicated with green lines. Asterisks refer to bifurcation points; white arrows indicate side branches. **(E,F)** Zoom images of a side branch **(E)** and a bifurcation point **(F)** of the organoid depicted in **(C)**. *Cdh1-CFP* is shown in cyan; smooth muscle actin labeling is shown in magenta. Scale bars represent 100 μm.

To study morphological changes, such as branching morphogenesis, it is important to strictly define and standardize the measurements. Therefore, we defined several parameters to describe the branched mammary organoid structures under different conditions:

- *Organoid length* (or *elongation*) was measured in the direction of maximum elongation (Figure 3A).
- *Organoid area* was measured using the maximum projection (Figure 3A) in ImageJ software (NIH).^1^
- *Organoid thickness or depth* was measured using the LasX software depth coding function (Figure 3B).
- *Organoid core* was defined as the primary structure of the organoid from which the branches or buds initiate. This could be either a sphere-like structure in the center of a budding organoid or the primary branch of a branched organoid.
- *Organoid buds* and *branches:* As the novelty of our model is the induction of both elongation and branching, it is critical to discriminate between “branches” and “buds.” Morphologically, branches can be distinguished by their duct-like elongated structure with a terminal tip, while buds are characterized by their semi-spherical shape developing directly from the core (or from a branch; Figure 2B, arrows indicate branches and asterisks indicate buds). For quantitative analysis, we defined a length threshold to discriminate between buds and branches, defining branches as structures with a length >70 μm and buds as structures with a length <70 μm. To measure *branch/bud length*, the organoid origin was first defined as follows: the perimeter of the spherical core or primary branch of the organoid was determined (Figures 3C,D, yellow lines).
- *Branch length* was derived as follows: for primary branches the distance from branch end to organoid origin was measured (Figures 3C,D, red lines), while for higher-level branches the distance from the tip/end of the branch to the line defining the previous level branch was determined (Figures 3C,D, green lines).
- *Branch levels* were determined as follows: a branch extending from the side of a previously formed branch was defined as a next branch level (Figure 3E). If two branches developed in parallel following a bifurcation event, they were defined as same level branches, but one level higher than the branch from which they developed (Figure 3F).

## Materials and Equipment

### Reagents

Mouse mammary gland digestion and organoid plating in BME:

- Phenol-red free Dulbecco’s Modified Eagle Medium (DMEM)/F12 Nutrient Mixture (Gibco^™^ 11039-021)
- Penicillin–Streptomycin (Pen-Strep, Gibco^™^ 151140-122)
- Collagenase type IV from *Clostridium histolyticum* (Gibco^™^ 17104-019)
- 0.25% Trypsin–EDTA 1X (Gibco^™^ 25200-056)
- Insulin-Transferrin Selenium (ITS) solution 100X (Gibco 41400-045)
- Fetal Bovine Serum (FBS; Gibco^™^ 10270-106)
- Deoxyribonuclease I (DNase I, Invitrogen 18047-019)
- Bovine Serum Albumin (BSA) Fraction V (Roche Diagnostics 10774111103)
- Red Blood Cell Lysing Buffer (Sigma-Aldrich R7767)
- Phenol red-free TrypleE^™^ Express (Gibco^™^ 12604-013)
- Cultrex Reduced Growth Factor Basement Membrane Extract (BME) Type 2 (R&D Systems 3532-005-02) or, as alternative, Growth Factor Reduced Matrigel (Corning 354230)

Organoid plating in BME:collagen I mixed gels:

- Rat tail Collagen I (Corning 354236)
- 10X Low Glucose DMEM (Sigma-Aldrich 2429)
- 0.5N NaOH

Factors for medium supplementation:

- Y-27632 (p160ROCK inhibitor, PeproTech 1293823)
- Fibroblast Growth Factor 2 (FGF2, PeproTech 100-18B)
- Epithelial Growth Factor (EGF, PeproTech 3165-09)

Whole-mount organoid staining:

- 4% Paraformaldehyde (PFA, AlfaAesar 47347)
- Normal Goat Serum (NGS, Thermo Fisher, Gibco^™^ 16210072)
- Triton X-100 (Sigma-Aldrich T8787)
- Primary antibodies: anti-α-smooth muscle actin (SMA), mouse-IgG2a (Sigma-Aldrich A5228, 1:600), anti E-cadherin monoclonal Decma1, rat (Thermo Fisher 14-3249-82, 1:500), anti-Estrogen Receptor alpha (ERα), rabbit (13258S Cell Signaling, 1:100), anti-Progesterone Receptor (PR), rabbit (MA5-14505 Thermo Fisher, 1:200)
- Secondary antibodies: goat anti-mouse IgG2a Alexa-647 (Thermo Fisher A21241, 1:500), donkey anti-rabbit Alexa Fluor 568 (TermoFisher A10042, 1:500) and donkey anti-rat Alexa-488 (Thermo Fisher A21208, 1:500).
- Vectashield Antifade Mounting Medium (Vector Labs, H-1400)
- 4′,6-diamidino-2-phenylindole (DAPI, Thermo Fisher D21490 1:500)

### Preparation of Solutions and Media

1. *Collagenase solution* (10 ml per mouse, prepared fresh): combine 9.5 ml of DMEM/F12 supplemented with 5% Pen-Strep, 500 μl of FBS, 5500 units of collagenase type IV (20 mg of collagenase IV powder from a 275 U/mg stock), 8 μl of 0.25% Trypsin–EDTA, and 5 μl of ITS. *Store at 4°C until usage on the same day*.
2. *BSA solution* (2.5%): add 1.25 g of BSA to 50 ml of PBS. Shake the tube by hand until the powder is completely solubilized and filter sterilize through a 0.22 μm filter into a new 50 ml falcon. *Store at 4°C*.
3. *Neutralized collagen I:* prepare the desired final volume of neutralized collagen I according to the following formula. The step-by-step procedure is reported in detail in steps 1–6 in sub section “Preparation of Pre-assembled Collagen I”. It is advised to prepare a minimum of 500 μl to ease mixing and avoid formation of air bubbles that could impair proper collagen assembly.

**Table 1:** 

Total volume of neutralized collagen (μl)	500	1,000
Collagen I stock (μl)	434	868
10X DMEM low glucose (μl)	50	100
0.5 N NaOH (μl)	2.5	5
4. *Basic Organoid Medium (BOM):* Remove 5 ml of DMEM-F12 from a 500 ml bottle and add 5 ml of Pen-Strep (5,000 U/ml of penicillin and 5,000 μg/ml of streptomycin). Supplement BOM with 1X ITS and, additionally, with 3 μM of Y-27632 (Y-BOM) for the first 24–48 h after the first plating and after each split to prevent cell death by anoikis in the BME dome.
5. *Alternation Treatment Basic Organoid Medium:* supplement BOM, in alternation, with 2.5 nM of FGF2 for 3 days (F-BOM) and 2 nM of EGF (E-BOM) for 4 days. In addition, supplement BOM with 3 μM of Y-27632 (Y-BOM) for the first 24 h of the alternation treatment. The alternation treatment timeline and factor supplementation are depicted in Figure 1G.

### Tools

- Micro-dissecting scissors, size 4 1/4 in. stainless steel straight, sharp point (Sigma-Aldrich, S3146)
- Jewelers forceps, Dumont No. 5,L 4 1/4 in., Inox alloy (Sigma-Aldrich F6521-1EA)
- Swann-Morton^™^ Stainless Steel Surgical Scalpels (normal blade - for mouse dissection, VWR 233-5363)
- Polystyrene Petri Dish (Fisher Scientific AS5052)
- Centrifuge tubes, Falcon 15 ml PS material (VWR 734-0450)
- Fisherbrand^™^ Sterile PES Syringe Filter – 0.2 μm (Fisher Scientific 15206869)
- Ice bucket
- 24-well plate Corning^™^ Costar^™^ Flat Bottom Cell Culture Plates (Fisher Scientific 10732552)
- Glass bottom Ibidi plates (μ-Slide 8 well Glass Bottom, Proxylab 80827)
- Microscope Cover Glass 22×50 mm (Duran Group)

### Equipment

- Orbital shaker (Stuart SSL4 Rocker)
- Incubator Shaker (Eppendorf Innova44 Incubator Shaker)
- Isoflurane station (Rothacher Medical)
- Centrifuge (Eppendorf centrifuge 5804R)
- pH meter (Mettler Toledo)
- Leica SP8-DIVE inverted confocal microscope equipped with an InsightX3 multiphoton laser (Spectra Physics), as well as a 405, 488, 561, and 638 nm laser
- 25× water objective (HC FLUOTAR VISIR), NA 0.95, working distance 2.4 mm.

### Mice

All mice were females housed at the KU Leuven Animal Facility. All experiments involving animals were performed in accordance with the Institutional Animal Care and Research Advisory Committee of the KU Leuven. Primary mammary gland organoids were prepared by harvesting mammary glands from 8- to 18-week-old nulliparous FVB/NJ *Cdh1-CFP* female mice (Snippert et al., 2010), hereafter also referred to as Ecad-CFP. *Cdh1-CFP* organoids were used for protocol development, endpoint branching analyses, and multiphoton imaging. Primary mammary gland organoids derived from C57/BL6 female mice were used to test the reproducibility of our novel branching protocol in a different genetic background.

## Results

**Figure 4:**
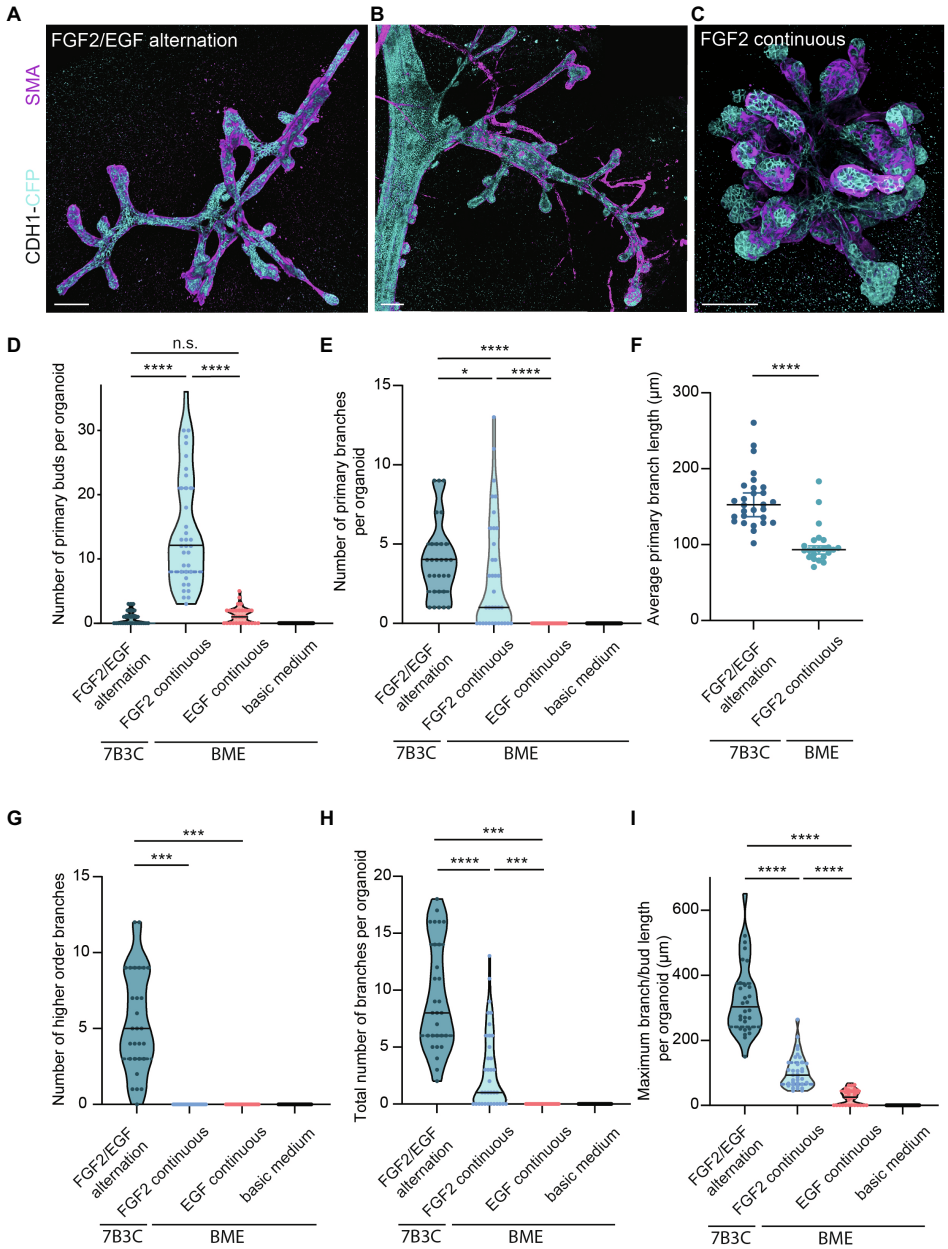
Comparison of branching parameters between different mammary organoid culture conditions. **(A)** Representative 3D rendering of a branched mammary organoid obtained after 14 days in 7B3C with FGF2/EGF alternation treatment. **(B)** 3D rendering of a whole-mount mammary gland derived from a 10-week-old *Cdh1-CFP* female mouse. Note the morphological similarities with the mammary organoid depicted in (a). **(C)** Organoid grown in BME with medium supplemented with FGF2, resulting in the formation of budding structures. *Cdh1-CFP* is shown in cyan; smooth muscle actin labeling is shown in magenta. Note that not all buds are covered with myoepithelial cells. Scale bars represent 100 μm. **(D)** Quantification of the number of primary buds per organoid, defined as structures <70 μm, in the different culture conditions. Black lines indicate median value, and dotted lines indicate the 25th and 75th percentile. **(E)** Quantification of the number of primary branches per organoid defined as structures >70 μm. Black lines indicate median value and dotted lines indicate the 25th and 75th percentile. **(F)** Comparison of average primary branch length per organoid between 7B3C alternation treatment conditions and BME with continuous FGF2 supplementation. Black lines indicate median value, and colored lines indicate 95% confidence interval. **(G–I)** Quantification of number of higher order branches **(G)**, total number of branches **(H)**, and maximum branch length **(I)** per organoid in the indicated culture conditions. All parameters were quantified at day 18 of culture for all conditions; data represent >30 organoids per condition derived from three experimental replicates. Significance was tested using Mann–Whitney test, ^*^p ≤ 0.05, ^***^p ≤ 0.001, ^****^p ≤ 0.0001.

We report a novel mammary organoid culture protocol that combines mixed BME:collagen I gels (7B3C) with an alternating treatment with FGF2 and EGF following a specific timeline (Figure 1G). Our mammary organoid culture protocol results in highly complex and branched organoids (Figure 4A) resembling the *in vivo* branched morphology of the mammary gland (Figure 4B). In the optimized timeline, FGF2 is supplemented in the medium from the start of the branching protocol when small spheres are seeded in 7B3C (Figure 2B, first panel, Supplementary Figure S1F). One day later, the medium is exchanged with fresh F-BOM, and subsequently alternating medium changes are performed with E-BOM and F-BOM resulting in growth factor pulses every third or fourth day, respectively (Figure 1G). Sphere-shaped organoids respond to these culture conditions with characteristic time-specific morphological changes. At day 3–4 of culture, buds emerge from the central organoid core, similarly to classical culture conditions in pure Matrigel/BME supplemented with FGF2 (Figure 2B, second panel). At day 7–8, after the first cycle of FGF2/EGF alternation, elongated primary branches are formed, and secondary branches start to emerge (Figure 2B, third panel). These secondary branches develop either by primary branch bifurcation or as side branches of previously formed branches (Figures 3E,F). From day 10 onwards, the morphological variability among different organoids increases, where some organoids grow complex structures, whereas others remain rather simple. However, at day 14–15 of culture, we generally observed elongated primary and secondary branches and shorter tertiary branches in the majority of the organoids that initially showed branching potential (Figure 2B, last panel). It is of note that we managed to extend organoid culture up to 25 days, enabling to follow biological processes occurring over a longer time scale.

Next, we characterized the morphology of our branched organoids and compared their characteristics with the current commonly used mammary organoid conditions. To this end, we cultured organoids in BME supplemented with BOM, F-BOM, or E-BOM (refreshed every 3 days), and compared their morphology and branching capacity with the organoids obtained in 7B3C gels using FGF2/EGF alternation treatment (Figures 4A,C–I). Mammary organoids grown in BME and BOM or E-BOM remained spherical and devoid of buds or branches (Figures 4D–I). Mammary organoids cultured in BME with continuous FGF2 supplementation resulted in a highly budded phenotype over an 18-days culture period (buds defined as structures <70 μm; Figures 4D,E). Some buds extended >70 μm which we considered as branches; however, their length was still significantly smaller compared to primary branch length in the alternation treatment induced branched organoids (Figures 4E,F). Importantly, many buds in the BME with continuous FGF2-supplementation were devoid of myoepithelial cells and therefore not representative of the *in vivo* bi-layered nature of the mammary ducts (Figure 4C). Previous studies have exploited this setup as a model of branching morphogenesis, albeit as short-term cultures up to 10 days (Zhang et al., 2014; Nguyen-Ngoc et al., 2015). Although rare bifurcation and side-branching events could be observed, none of the formed secondary buds extended to a length >70 μm to be considered as a secondary branching event (Figure 4G). Instead, and as previously suggested (Nguyen-Ngoc et al., 2015), these multilayered FGF2-induced buds may rather represent a simplified *in vitro* counterpart of pubertal TEBs. In contrast, organoids cultured in 7B3C supplemented with alternating FGF2 and EGF supplementation formed complex branched structures containing both primary and higher-level branches (Figures 4E–G), with all tips covered with a bi-layered epithelium consisting of an outer layer of myoepithelial cells and an inner layer of luminal cells (Figure 4A). These branched mammary organoids not only contained more branches (Figure 4H), but also more elongated branches reaching up to 540 μm in length (Figure 4I).

**Figure 5:**
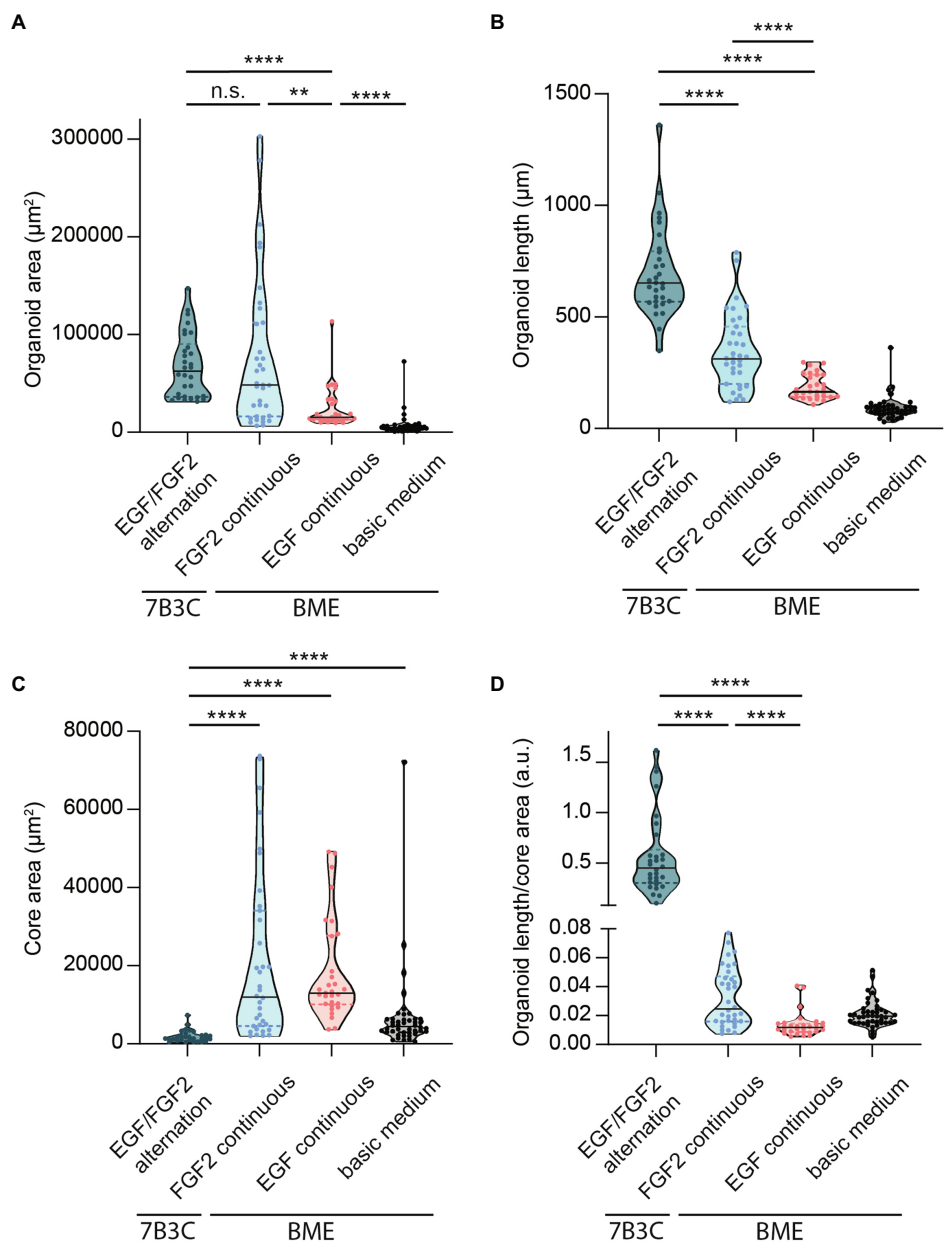
Quantification of mammary organoid size and morphology under different culture conditions. Quantification of mammary organoid area **(A)**, organoid length **(B)**, and organoid core size **(C)**. **(D)** Organoid length/core area was determined as a robust parameter to accurately describe the difference between complex branched organoids and hyperbranched or budding organoids. All parameters were quantified at day 18 of culture for all conditions; data represent >30 organoids per condition derived from three experimental replicates. Significance was tested using a Mann–Whitney test, ^**^p ≤ 0.01, and ^****^p ≤ 0.0001.

Besides differences in branched morphology, we also identified changes in total organoid size between the different culture conditions. Organoids grown in BME supplemented with F-BOM or in 7B3C in combination with alternation treatment reached a similar size and were both significantly larger compared to the other conditions, albeit with completely different morphologies (Figure 5A). Organoids grown in 7B3C with alternation treatment reached a larger length compared to all other conditions (Figure 5B), whereas organoids grown in BME with F-BOM reached an increased organoid core size (Figure 5C). These parameters clearly indicate a different driver of organoid growth in both conditions. Organoids grown in BME-FGF2 conditions typically developed a large organoid core and multiple wide buds leading to extended total organoid area. Instead, in the 7B3C-alternation conditions, the increase in organoid area was the result of the extensive network of elongated branches, while little or almost no organoid core was distinguishable after 18 days in culture.

Finally, to better describe the distinct organoid morphologies in one descriptive parameter, we determined the ratio between organoid length and core size (Figure 5D). Using this ratio, organoids with a branched morphology show median ratios >0.1, whereas large but hyper-budding organoids do not exceed a ratio of 0.08 (Figure 5D) resulting in a robust parameter to discriminate organoid size from organoid complexity.

To assess the similarities and differences between the morphology of the branched mammary organoids and the *in vivo* mammary gland, we compared several key parameters. In addition to the correct formation of an epithelial bilayer consisting of myoepithelial and luminal cells throughout the entire organoid (Figure 4A), we performed immunofluorescent labeling for ER and PR (Supplementary Figure S2). Indeed, a subset of the luminal cells throughout the entire organoid express ER or PR (Supplementary Figure S2A–D), similar to the expression pattern observed in the *in vivo* mammary gland (Supplementary Figure S2E). Next, we performed more detailed analysis of the branching patterns in the organoids and their *in vivo* counterpart. We quantified the number of buds and branches per ductal area and identified that the branched mammary organoids are more densely branched compared to the *in vivo* situation (i.e., branches are less elongated in the organoids compared to the *in vivo* mammary gland), resulting in a significantly higher number of branches or buds per mm^2^ (Supplementary Figure S3A,B). However, the ratio between buds and branches, which reflects the balance between elongation and termination of a branch, is similar between the branched organoids and the post-pubertal mammary gland (Supplementary Figure S3C). Finally, we compared the time scale of branching events in the branched mammary organoid model with the *in vivo* mammary gland counterpart. The murine mammary gland typically contains 30–35 branch levels (Hannezo et al., 2017), which together develop over a time scale of roughly 5 weeks. When comparing this time scale to our *in vitro* branched mammary organoids, which reach up to five branch levels after 20 days of FGF2/EGF alternation treatment, our organoid model of branching has a reduced branching and elongation speed. It is important to consider that *in vivo* mammary branching morphogenesis during puberty starts from an already organized rudimentary tree, whereas our organoid model starts from a sphere-shaped structure. Budding structures grow from these mammary spheres in the first 6–7 days, which could be considered a priming step before the program of alternated branching and elongation starts (Figure 2B). The majority of the branching events in our *in vitro* model occur between day 7 and day 17 of FGF2/EGF alternation treatment. Organoid branching and elongation slow down around day 14–17 and branch elongation is rare after day 17 of culture. Given these observations, we can rather define the time window between day 7 and 17 as the time during which branching events occur. In this scenario, both branching and elongation would occur, on average, at a speed of five branch levels over a timescale of 10 days. Although this is still a slightly reduced speed compared to the *in vivo* situation, we consider these dynamics sufficient to model branching morphogenesis in an *in vitro* system, especially when keeping in mind that our model only partially reproduces the complexity of *in vivo* mechanical and signaling cues.

Overall, we envision that the collection of these parameters enables a comprehensive description of mammary organoid morphology and will help in defining the impact of diverse culture conditions, such as growth factors, hormones, ECM compositions, or other cell types on morphological changes in mammary organoids. Our study suggests that the combination of the right ECM stiffness and growth factor complexity enables to induce an *in vitro* program of branching morphogenesis similar to the *in vivo* situation. We believe that our branched mammary organoid protocol represents a useful and reliable *ex vivo* tool to uncover the drivers and dynamics of *in vivo* branching morphogenesis and mammary gland remodeling.

### Troubleshooting

**Figure 6:**
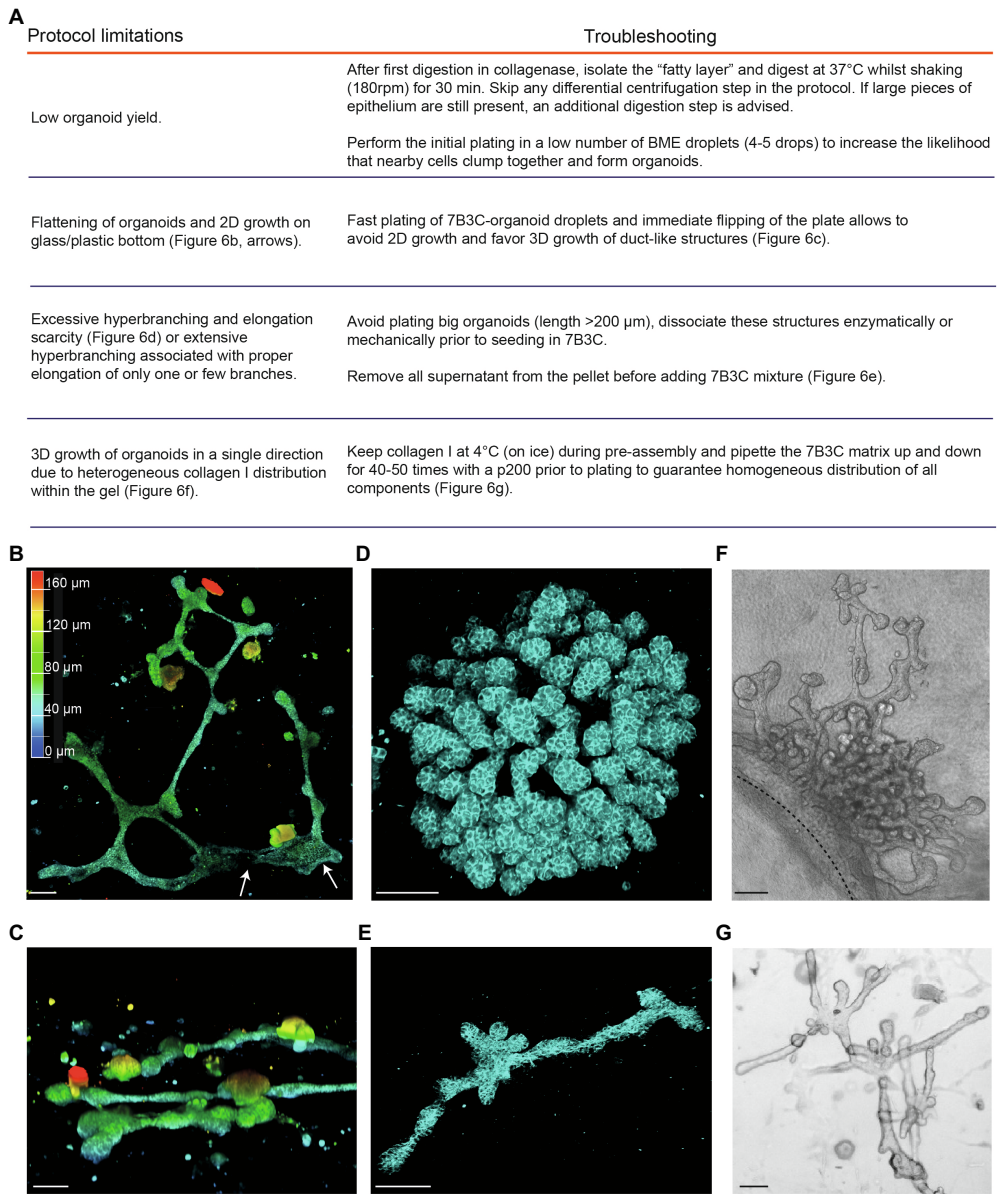
Limitations and troubleshooting of the mammary organoid branching protocol. **(A)** Overview of common pitfalls during the mammary branching organoid protocol. **(B)** Example of flattened organoid (3D rendering) on the bottom of the cell culture dish, top view. White arrows indicate 2D sheets of cells; colors indicate depth coding. **(C)** Confocal image of ductal organoid structures showing 3D growth, side view. **(D,E)** Confocal images (3D rendering) of a hyperbranched organoid with lack of branch elongation **(D)** and an elongated organoid after careful removal of all supernatant from the organoid pellet before adding the 7B3C mixture **(E)**. *Cdh1-CFP* signal in cyan. **(F)** Brightfield image showing local and uni-directional branching of an organoid with a large core area due to inhomogeneous collagen I distribution within the gel. **(G)** Brightfield image of a branched and elongated organoid obtained after thorough mixing of the 7B3C matrix prior to plating. Scale bars represent 100 μm.

Collectively, our organoid culture method using 7B3C in combination with FGF2/EGF alternation treatment is innovative in several ways. First, from the morphological point of view, the resulting organoids are not only branched and elongated, but also organized into miniaturized interconnected networks of duct-like structures. Second, our method provides the first *in vitro* model of crucial events during branching morphogenesis in the mammary gland, including bifurcation events and side branching, while at the same time the bi-layered nature of the ductal structures is maintained. Third, the resulting branched mammary organoid structures are optically accessible during the entire branching program, and our conditions allow to extend the organoid cultures to an average of 19 days, and up to 25 days. These characteristics implicate that our organoids are suited to study and follow the dynamics of branching morphogenesis and tissue (re)generation over a long-time scale at the single cell level. However, when compared to classical mammary organoid culture conditions in droplets of Matrigel, the generation of 7B3C gels and timed addition of growth factors is more complex, with multiple aspects that need to be precisely regulated to not compromise the phenotypic outcome. The protocol we developed is based on previously reported organoid and collagen pre-assembly assays (Nguyen-Ngoc and Ewald, 2013; Nguyen-Ngoc et al., 2015) but includes modifications that have been implemented based on empirical observations. Below, we report the most common pitfalls that could impair 3D culture and some possible troubleshooting strategies to overcome these impairments (a summary can be found in Figure 6A).

1. A common limitation we initially encountered was low organoid yield after harvesting and processing mammary tissues according to previously published protocols (Ewald et al., 2008; Nguyen-Ngoc et al., 2015). Especially in case of C57/BL6 donor mice, multiple mice were required to have enough organoids to perform statistically significant experiments. To improve organoid yield, we suggest the following procedures:
  - We observed that the fatty layer, which is typically discarded at protocol step in the section “Isolation of Primary Mammary Epithelial Organoids” (Figure 1B), still contained many epithelial pieces trapped in between the fat cells. To increase the organoid yield, we therefore recommend to perform an additional collagenase digestion step of the fatty layer, which results in a consistent increase in organoid yield.
  - When limited material is obtained, it is advised to first expand the organoids in smaller BME droplets to increase cell density.
2. After plating in 7B3C, we noticed that mammary organoids located very close to the bottom of the dish or to the collagen underlay (when present) tend to lose their 3D structure and spread out as 2D sheets of cells, or grow as extremely long duct-like structures (Figure 6B). Although some of these structures reflect *in vivo* branching morphogenesis in a quasi-2D fat pad to a certain extent, most 2D branched structures showed clear areas of 2D cell growth without any organization (Figure 6B). To prevent 2D organoid growth, we advise to plate the 7B3C droplets as fast as possible, followed by flipping of the plate and incubation upside-down at 37°C during gelation to facilitate the growth of 3D structures embedded in the middle of the 7B3C droplet (Figure 6C).
3. The parameter that has most impact on morphological outcomes of the branched mammary organoids is the 7B3C matrix stiffness. Therefore, it is of utmost importance to perform each step during 7B3C matrix preparation with great care. First, it is important to remove all supernatant from the organoid pellet before adding the 7B3C solution. This will avoid unwanted dilution of the 7B3C mixture with remnants of medium. We noticed that even a few tens of microliters could result in changes in matrix stiffness and formation of hyperbranched organoids that completely lack elongation, or result in organoids that develop only one or few long branches and multiple short buds (Figure 6D). In addition, it is crucial to allow collagen I pre-assembly for precisely 60–70 min to prevent a decrease in matrix stiffness (because of reduced fiber assembly) and as a consequence impaired branching and elongation.
4. The initial size of the organoids when seeded in 7B3C will affect the branched morphology. When larger than 200 μm (derived from large epithelial pieces), organoids have the tendency to hyperbranch, likely due to hindrance posed to elongation by the big organoid core (Figure 6D). Mechanical dissociation before plating is sufficient to reduce organoid size prior to plating into 7B3C, resulting in both branched and elongated structures (Figure 6E).
5. Heterogeneous fiber assembly within the 7B3C mixture will result in organoids with large core areas and localized hyperbranching (in the softer areas of the gel devoid of collagen I; Figure 6F). To promote organoid branching and elongation in all directions, it is important to keep the temperature of the 7B3C mixture as stable as possible during pre-assembly. Moreover, we advise to mix all the prepared solutions thoroughly prior to plating to avoid the formation of collagen-clumps that could act as a physical obstacle to proper branching and elongation of the organoids (Figure 6G).

## Discussion

Here, we present a protocol to generate branched mammary organoid structures. By alternating the addition of FGF2 and EGF, we obtain organoids with a complex morphology that resembles the branched structure of the adult mammary gland. Previously published protocols using similar mixtures of collagen I and BME supplemented with FGF2 already demonstrated the potential of organoids to form buds and branches (Ewald et al., 2008; Nguyen-Ngoc and Ewald, 2013; Nguyen-Ngoc et al., 2015), however, their complexity was still limited.

In an attempt to dissect the process of branching morphogenesis, we reasoned that two alternating processes are required to generate a branched morphology: branching and elongation. Among others, EGF and FGF2 were previously identified as key signals regulating mammary branching morphogenesis. FGF receptor 2 (FGFR2) is highly expressed in TEBs and FGFR2-null glands were shown to have reduced branching (Lu et al., 2008). FGF2, one of the ligands for FGFR2, was reported to be essential for ductal elongation, TEB cell proliferation (Zhang et al., 2014), and TEB maintenance (Parsa et al., 2008). In addition, FGF2 was previously shown to induce a simplified program of branching morphogenesis resulting in budding in primary organoid cultures in BME but failed to induce branch elongation (Nguyen-Ngoc et al., 2015). Hence, we identified EGF as a factor important for ductal growth and elongation. EGF secretion and EGF receptor (EGFR signalling) have a fundamental role in mammary gland ductal morphogenesis and glands from EGFR KO mice fail to develop beyond rudimentary structures (Sebastian et al., 1998). Additionally, mammary organoids cultured in BME with EGF supplementation show increased growth compared to the BOM condition but fail to form buds or branches. Another study in the branched submandibular gland showed that FGF signaling could sensitize epithelial cells to EGF at the initiation of branching morphogenesis (Nitta et al., 2009). Inspired by these recent findings, we designed our experiments using an alternated addition of FGF2 and EGF. Indeed, our method shows that alternation of EGF to FGF2 not only induces ductal elongation, but also result in the formation of a complex structure resembling the *in vivo* branched morphology. Interestingly, our data indicate that EGF can directly act on the mammary epithelial cells, which is in contrast with the current model of *in vivo* EGF signaling which proposes that branching morphogenesis is dependent on EGFR signaling in the stromal compartment which is lacking from the mammary organoid cultures (Ciarloni et al., 2007).

To better describe the branched organoid structures, and to compare different conditions, we defined several measures of branching complexity (Figure 4) and overall organoid morphology (Figure 5). Up to now, budding organoid structures induced by growth factor treatment have been arbitrary referred to as branches or buds without using a common criterium. This lack of definition renders it difficult to compare available mammary organoid culture conditions between each other and with the *in vivo* branched structure of the mammary gland. We noticed that the nomenclature in the field does not have any correlation with the morphology, where the terms “buds” and “branches” refer to different types of structures. Therefore, we sought to define the structures most commonly observed *in vivo* and *in vitro* to standardize the way they are measured, including a measurable definition of buds (structures <70 μm) and branches (>70 μm), a definition of branch levels, as well as a discrimination between organoids with and without a core area. The branching parameters were derived from empirical observations and verified on different batches of organoids derived from different mouse strains. Important to note is that buds and branches in the mammary organoids were measured after a defined culture period. It may be that some of the buds would elongate further into branches if the culture was kept over a longer time period. Using these measurements, we identified that both the number of primary buds and the number of primary and higher-level branches can be used to discriminate between the hyper-budding core-retaining phenotype and the simultaneously branched and elongated organoids. Moreover, a simple parameter, such as the ratio between organoid length and core area, could be easily implemented to distinguish simple organoid structures from branched organoids resembling the *in vivo* branched morphology.

Interestingly, side-branching/bifurcation and elongation occurred to different extents during both EGF and FGF2 treatment time frames. During the EGF stimulation, branch elongation was mostly observed, whereas during the FGF2 periods, branching events were more apparent. The development of side branches has never been previously reported and characterized in murine mammary gland organoid models. We observed that side branches developed only after the first cycle of FGF2/EGF alternation, while they very rarely developed in FGF2 continuous conditions. The need of a timed addition of growth factor supplementation to obtain proper elongation and branching events suggests that the signaling events regulating branching morphogenesis may work in waves of activation of downstream signaling pathways. Indeed, such models have been previously proposed in other organs such as the lung, the submandibular gland, and in mammary acini and spherical organoids (Nitta et al., 2009; Ender et al., 2020; Rabata et al., 2020; Sumbal et al., 2020; Gagliardi et al., 2021). Further experiments using refined models for branching morphogenesis, including branched mammary organoid models, will be necessary to fully dissect the signaling events driving branching, bifurcation, and elongation in the mammary gland.

## Data Availability Statement

The original contributions presented in the study are included in the article/supplementary material, further inquiries can be directed to the corresponding author.

## Ethics Statement

The animal study was reviewed and approved by the Institutional Animal Care and Research Advisory Committee of the KU Leuven.

## Author Contributions

LM and CS conceptualized and designed the study. MC and SH designed, performed, and analyzed the experiments supervised by LM and CS. MC, LM, and CS wrote the manuscript. The manuscript was reviewed and approved by all authors.

## Funding

This work was supported by an EMBO postdoctoral fellowship (ALTF 1035-2020 to CS).

## Conflict of Interest

The authors declare that the research was conducted in the absence of any commercial or financial relationships that could be construed as a potential conflict of interest.

## Publisher’s Note

All claims expressed in this article are solely those of the authors and do not necessarily represent those of their affiliated organizations, or those of the publisher, the editors and the reviewers. Any product that may be evaluated in this article, or claim that may be made by its manufacturer, is not guaranteed or endorsed by the publisher.

## Footnotes

^1^ https://imagej.nih.gov/ij/
