# Supplementary material for: A Mammary Organoid Model to Study Branching Morphogenesis
Source: Front Physiol. 2022 Mar 16;13:826107. doi: 10.3389/fphys.2022.826107 (PMC8988230; doi:10.3389/fphys.2022.826107)
Supplement: Supplementary file 1 [file Data_Sheet_1.docx]

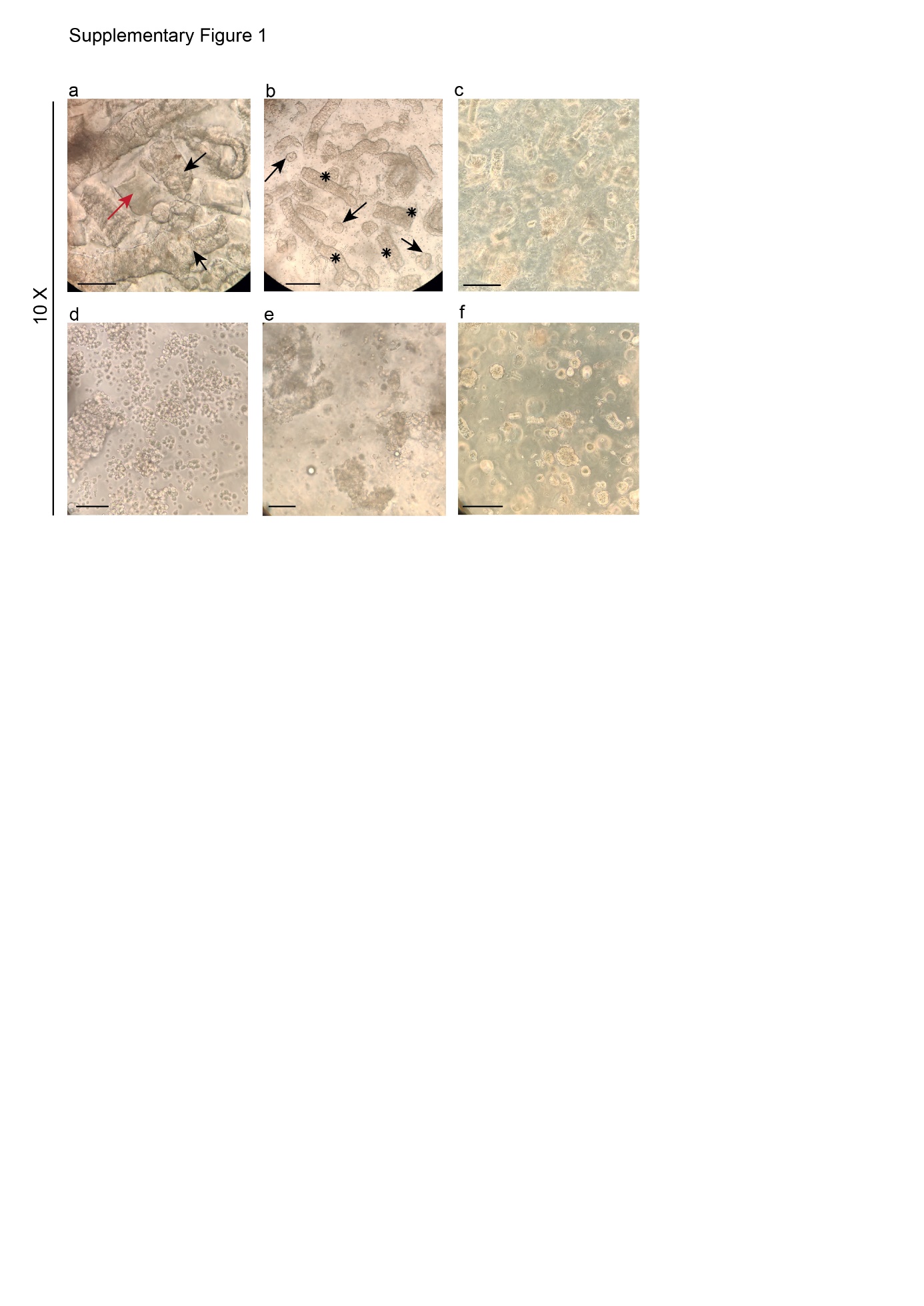


**Supplementary Figure 1 | Collagenase digestion of mammary glands to obtain mammary epithelial fragments. (a)** Representative brightfield image of under-digested mammary epithelial fragments (black arrows) and the presence of muscle fibers (red arrow). **(b)** Representative brightfield image of a partially digested pool of large mammary epithelial fragments (asterisks) and small epithelial pieces (black arrows). **(c)** Brightfield image of a well-digested mixture of epithelial fragments ready for plating in BME droplets. **(d)** Image depicting an over-digested preparation of cells. **(e)** Clumps of mammary epithelial cells and fragments that require mechanical dissociation prior to plating into BME droplets. **(f)** Representative brightfield image of mammary epithelial organoids, as spheres, plated in BME droplets. Scale bars represent 100 µm.­­­


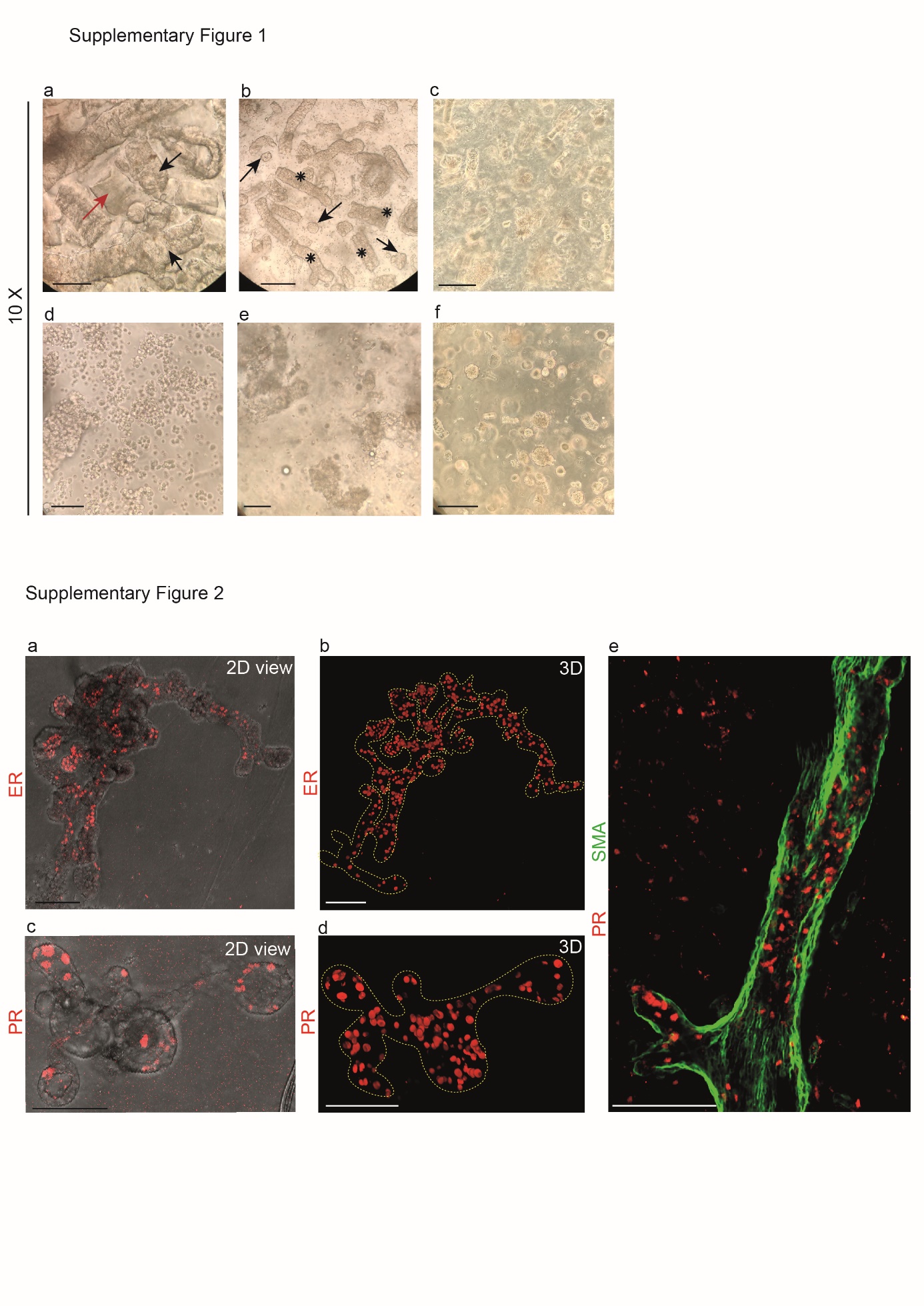


**Supplementary Figure 2 | Characterization of hormone receptor expression in organoids grown in 7B3C under FGF2/EGF alternation treatment. (a)** 2D brightfield view of the morphology of an organoid merged with immunofluorescent signal for Estrogen Receptor (ER, in red) at day 21 of FGF2/EGF alternation treatment. **(b)** 3D immunofluorescent view of the organoid in (a)**. (c)** 2D brightfield view of the morphology of an organoid merged with immunofluorescent signal for Progesterone Receptor (PR, in red) at day 17 of FGF2/EGF alternation**. (d)** 3D immunofluorescent view of the organoid in (c). Dashed yellow lines in the 3D views represent the reconstruction of organoid shape based on the brightfield images. **(e)** Representative image of PR^+^ expression pattern in ducts and tips from a cryo-section of a post-pubertal mammary gland stained for α-SMA (in green) and PR (in red). PR^+^ cells (and ER^+^ cells, data not shown for *in vivo* mammary gland) are homogeneously distributed in ducts and tips in the *in vivo* gland and in the branched organoids. Scale bars, 100 µm.


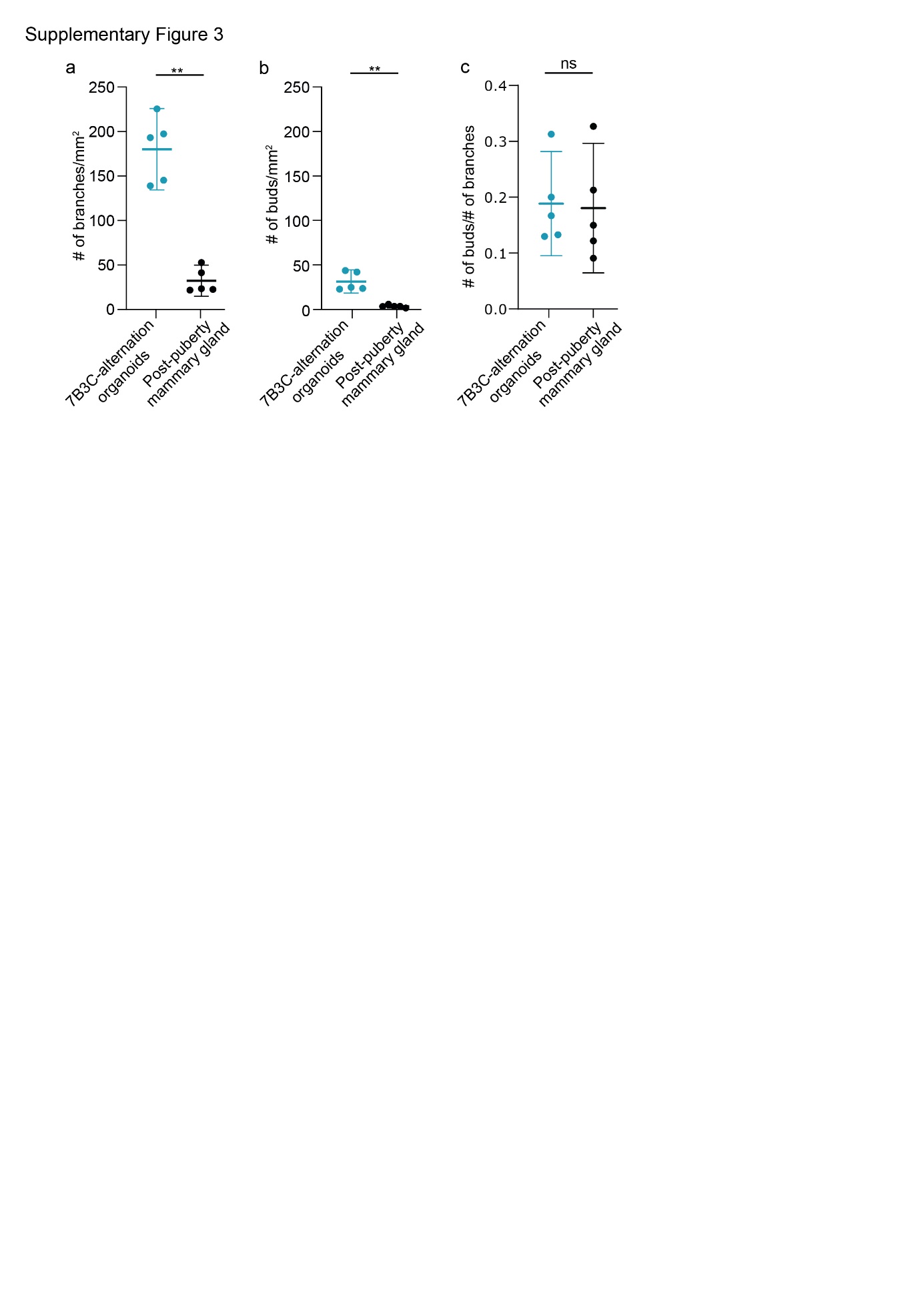


**Supplementary Figure 3 | Quantification of branching pattern in the post-pubertal gland and in branched organoids.** To compare the branching pattern of our *in vitro* organoids to the *in vivo* mammary gland, the number of branches/mm^2^ **(a)** and the number of buds/mm^2^ **(b)** were derived by counting the number of branches and buds in five regions of a mouse post-pubertal gland and in five organoids grown in 7B3C gels at day 14 of FGF2/EGF alternation treatment. The number of branches and buds was normalized to ductal area for the *in vivo* post-pubertal gland or to total organoid area. **(c)** Ratio between the number of buds and the number of branches. Graphs report the mean values and the associated 95% confidence interval. Significance was tested using a Mann-Whitney test, **P≤0.01.
